# Identifying Sources of Moral Distress Amongst Critical Care Staff During the Covid-19 Pandemic Using a Naturalistic Inquiry

**DOI:** 10.1177/23779608231167814

**Published:** 2023-04-04

**Authors:** Margaret Scott, Rachel Wade, Guy Tucker, John Unsworth

**Affiliations:** 1 6072 Northumbria Healthcare NHS Foundation Trust, Northumberland, UK; 2 South Tyneside and Sunderland NHS Foundation Trust, Sunderland, UK; 3 Northumbria University, Newcastle upon Tyne, UK

**Keywords:** moral distress, pandemic, critical care, sources

## Abstract

**Introduction:**

Moral distress can have a significant impact on the mental health and well-being of practitioners. Causes of moral distress in critical care have been identified as futile treatment, conflict between family members and staff, lack of resources, and dysfunctional teams.

**Objectives:**

This study explores the sources of moral distress during the COVID-19 pandemic and the meaning that staff attached to these events. The study aims to examine whether the sources of moral distress are similar, or different, to those that commonly occur in critical care departments.

**Methods:**

Naturalistic inquiry using semi-structured individual interviews with 17 participants drawn from nursing (*n* = 12), medicine (*n* = 3), and the allied health professions (*n* = 2). The interviews were recorded and transcribed verbatim. The transcripts were analyzed using reflexive thematic analysis.

**Results:**

The results suggested that while there were some similar sources of moral distress including caring for dying patients and not being able to provide the usual standard of care, the nature of the disease trajectory and frequency of death had a significant impact. In addition, the researchers found that providing care which was counter-intuitive, concerns about the risks to the staff and their families and the additional burdens associated with leading teams in times of uncertainty were identified as sources of moral distress.

**Conclusion:**

This study explored the potential sources of moral distress during the pandemic and the meaning that practitioners attached to their experiences. There were some similarities with the sources of moral distress in critical care which occur outside of a pandemic. However, the frequency and intensity of the experiences are likely to be different during a pandemic, with staff describing high volumes of deaths without family members present. In addition, new sources of moral distress related to uncertainty, counter-intuitive care and concerns about personal and family risk of infection were identified.

## Introduction

Moral distress and moral injury are known to have a significant impact on health professionals leading to burnout, depression, and in some cases to post-traumatic stress disorder (Williamson et al., 2021). Moral distress occurs when a health professional experiences a situation which conflicts with the morally accepted norms for care delivery. The concept was first conceptualized in healthcare by Jameton (1984) who described it as a psychological discomfort that occurs when a person is unable to complete what they believe to be an ethically responsible action due to internal or external factors. Moral distress can threaten practitioners’ mental health and well-being as they grapple with the moral dilemmas presented by the practice scenario. The negative impact of continued exposure to morally distressing situations can result in moral injury with longer-term impacts on the practitioner including burnout, anxiety, and depression (Rushton, 2018). Moral distress is the immediate result of being faced with a morally challenging situation. Cartolovni et al. (2021) describe how the distress may continue for hours or days after the incident but often resolves. Repeated or severe violations of a moral nature may leave a moral residue which can accumulate into moral injury (Atuel et al. 2021).

Many studies (Colville et al., 2019; Corley, 1995; Mealer & Moss, 2016) into moral distress have been conducted in the critical care setting. Critical care is regarded as an environment where moral dilemmas may play out more frequently than in other settings because of potentially futile treatment, conflict around decisions to end life support, and because of the acuity of patients (Mealer et al., 2007). Grailey et al. (2021, p. 1) describe how “the intensive care unit can be a busy relentless and stressful working environment.” Staff are required to care for the sickest population of patients within the hospital, often in rapidly changing clinical scenarios and emergencies. They must also contend with constant pressure to manage patient flow within the constraints of capacity.

Prior to the COVID-19 pandemic moral distress in critical care had received considerable attention enabling the identification of numerous potential sources of distress (Corley, 2002). The sources of moral distress include potentially futile treatment (Mealer & Moss, 2016), inadequate communication during end-of-life care (McAndrew et al., 2018), false hope (Wiegand & Funk, 2012), inadequate staffing (Morley et al., 2019), lack of resources (Walton, 2018), and conflict with physicians (Choe et al., 2015).

The COVID-19 pandemic placed unrelenting pressure and demand on healthcare systems across the world, leading to an unprecedented number of hospitalizations and deaths. The United Kingdom (UK) saw successive ways of infection with increased hospitalizations and a high number of admissions to critical care.

While pandemics are thankfully relatively rare there are more than 100,000 outbreaks of infectious disease globally (Word Health Organisation, 2019). This study explores sources of moral distress during the Covid-19 pandemic comparing this to existing knowledge about moral distress in critical care more generally. Understanding the differences in sources of moral distress during a pandemic may inform future practice during infection outbreaks and for future pandemics.

## Review of the Literature

Health professionals working within critical care are exposed to numerous physical and psychological stressors which can result in moral distress. Moral distress has been associated with decreased job satisfaction problems with turnover, heightened states of psychological distress and burnout (Hiler et al., 2018).

Hiler et al. (2018) explored the severity of moral distress among critical care nurses in the United States. Participants were invited to complete the moral distress scale alongside a demographic questionnaire. A total of 328 critical care nurses completed the survey. The research revealed high levels of moral distress when nurses perceived that care was futile. Higher levels of moral distress occurred when the wishes of the patient's family to continue life support were followed even though the nurse believed it was not in the patient's best interest. Analysis of the relationship between moral distress and the practice environment suggested that as the practice environment deteriorates in terms of work pressure and team working moral distress is high. Participants reported that inadequate staffing and a lack of resources increased moral distress.

Hines et al. (2021) studied trends in moral injury and distress and resilience factors among healthcare workers at the beginning of the COVID-19 pandemic using surveys over a three-month period. The researchers identified that while moral injury remained stable moral distress declined over the period. Protective factors include a supportive workplace environment. The degree of moral distress was not affected by any baseline occupation or other resilience factors. However, fatigue and poor sleep at baseline predicted greater moral distress in those individuals.

Research related to sources of moral distress has sought to measure the frequency and intensity of moral distress rather than explore in detail the factors which contribute to this and how they interact and play out in clinical settings.

Emmamally and Chiyangwa (2020) conducted a descriptive survey using a 21-item Moral Distress Scale. The moral distress composite scores for the 74 critical care nurses in South Africa revealed a min composite moral distress score of 112.12. Further analysis revealed that female respondents experienced higher distress scores than males and there was also a relationship between composite scores and increases in age and years of service. The most frequently encountered sources of moral distress in critical care were related to following the family's wishes to continue life support even when this was not in the patient's best interests: continuing care when it was regarded as futile, carrying out unnecessary tests and treatments and dealing with family members in relation to discussing prognosis and future care.

Karanikola et al. (2014) examined the levels of moral distress among staff working in intensive care in Italy and in particular explored the association between moral distress and nurse–physician collaboration, autonomy, professional satisfaction, intention to resign, and workload. A cross-sectional correlational design with a sample of 566 Italian intensive care nurses was surveyed using self-reported questionnaires. The intensity of moral distress was 57.9 (scale range 0–84 and the frequency of occurrence based on the same scale was 28.4). The severity of moral distress was associated with nurse–physician collaboration and dissatisfaction with care decisions.

Lake et al. (2021) conducted a study exploring hospital nurses’ moral distress and mental health during the COVID-19 pandemic; the research was conducted during the first wave of the pandemic. The research utilized a cross-sectional correlational design and surveyed nurses from two academic medical centers in the United States. A total of 338 registered nurses completed the survey representing a 43% response rate. Amongst the respondents, 73% had cared for at least one COVID-19 patient and 71% cared for COVID-19 patients every day. The most commonly reported causes of moral distress were accessing Personal Protective Equipment alongside issues with communication from hospital management. Respondents also reported distressing situations related to the risk of transmission of the infection to their family members and caring for patients without their family members present. These factors occurred occasionally with moderate levels of distress.

Romero-Garcia et al. (2022) conducted a study of moral distress and its emotional impact on intensive care unit staff during the COVID-19 pandemic. The study utilized a cross-sectional survey-based design with the survey distributed to 434 permanent and temporary intensive care staff during the first wave of the pandemic. The research has found that the most common sources of moral distress during the pandemic were related to the patient and the family as well as resources and the organization. The researchers found that temporary staff who had been redeployed from other units had higher scores in relation to moral distress than permanent staff as well as a greater intention to leave their current position.

The literature review suggests that sources of moral distress among staff in critical care during the pandemic may have been different to the commonly cited sources prior to the pandemic. This study explores the sources of moral distress and the meaning that staff attached to these events. The study specifically seeks to:

1. Explore the sources of moral distress experienced by critical care staff during the Covid-19 pandemic.
2. Identify the meaning that critical care staff attached to the sources of moral distress and any factors which provide support when dealing with such challenges.

## Methods

### Design

This study aims to examine sources of moral distress and the meaning that staff working in critical care attach to their experiences. The desire to examine the meaning staff attached to their experiences of morally distressing aspects of care delivery meant that a research approach moving beyond description was needed (Creswell, 2014). For this reason, the study utilized a constructivist paradigm as the philosophical basis of the research. More specifically, the study utilized naturalistic inquiry in order to study the phenomena of moral dilemmas which gave rise to moral distress within the context in which it occurred (Guba & Lincoln, 1982). Within naturalistic inquiry, there is an acceptance that the interactions between participants and the researcher are mutually beneficial in constructing meaning (Cutler et al., 2021).

The method used in this research was semi-structured interviews using a series of open questions designed to invite participants to share their experiences and how they had subsequently made sense of each situation. Table 1 provides examples of the guiding research questions for the semi-structured interviews.

**Table 1.** Guiding Questions for the Semi-Structured Interviews.

The UK has a two-week window to prepare before pandemic wave 1 - Tell me about your experiences during that pre-wave 1 period? - Tell me how you felt? - What impact did news reports and information from Northern Italy have on you and your colleagues?
2. Describe your experiences of caring for patients during the pandemic?
3. How did you feel as the pandemic progressed?
4. Tell me what you personally learnt during the pandemic?
5. What impact did working in critical care during the pandemic have on you and others?

### Research Questions

The research sought to answer the following research questions:

1. What were the sources of moral distress experienced by critical care staff during the Covid-19 pandemic?
2. How did the sources of moral distress in critical care during the pandemic differ from those reported in the literature prior to the pandemic?
3. What meaning did critical care staff attach to the sources of moral distress and what approaches to support staff were deemed as helpful when facing such challenges?

### Sample

The research involved collecting data from a range of critical care staff including registered nurses, healthcare assistants, student nurses, medical staff, and allied health professionals. A total of 17 interviews were conducted between the second and third UK wave of the pandemic (March–June 2022).

### Inclusion/Exclusion Criteria

The inclusion criteria for the sample were staff working in critical care caring for patients with Covid-19 for a minimum of 4 weeks. The exclusion criteria were those staff caring for non-Covid-19 patients and those who had worked in the department for less than 4 weeks.

### Ethical Review

Exploring potentially distressing topic areas during research interviews required the researchers to conduct interviews with a high degree of sensitivity. In addition, the researchers ensure that support was available from the Trusts Psychological Services for anyone who needed additional support.

The study obtained ethical approval from the NHS Research Authority (294192) and from the University of Northumbria Faculty Ethics Committee. In addition, NHS Research and Development site approval was obtained prior to recruitment and data collection.

### Data Collection Setting

The setting for this study was a large critical care department in an emergency care hospital in the North of England. The department is built around a central hub with nine intensive care beds on one side and nine intensive care beds on the other side. This allowed the creation of specific Covid-19 areas or “Pods.” The department is able to flex between Level 2 (High Dependency care) and Level 3 (Critical Care requiring mechanical ventilation and/or support for two or more organs). A further surge critical care unit with six beds (Pod C) was created in the operating room staffed by operating department staff. This was for critical care patients other than those infected with Covid-19. Interviews were conducted in a quiet room within the critical care department between waves of infection.

### Data Analysis

The interviews were recorded and transcribed verbatim. The transcripts were analyzed using reflexive thematic analysis (Braun & Clarke, 2019) between July and November 2022. This reflexive approach fits well with a naturalistic inquiry as the researchers themselves contribute to developing meaning through the process of research and data collection. Within reflexive approaches to thematic analysis, the process starts with data familiarization, moving then to coding and the identification of themes, refining and reviewing themes and finally producing the report. In reflexive thematic analysis, there is no attempt to pre-define themes rather progression through the analysis will tend to facilitate further familiarity with the data, which may in turn result in the interpretation of new patterns of meaning (Byrne, 2022). Coding involves both the semantic coding of words and phrases and then latent coding where themes are identified through a deeper level of analysis (Braun & Clarke, 2020). Following the identification of initial themes broader themes are developed and then a working definition of each theme is produced. Table 2 provides an overview of this process from initial codes through to themes and their definition.

**Table 2.** Data Analysis Revealing Codes, Initial Themes, Themes, and Definitions.

	Examples of initial codes	Initial Themes	Theme	Definition
1	Initial guidance Opposite to normal Complications Too much guidance Does not make sense Totally unfamiliar Not knowing Best practice Expectations Disease trajectory	Guidance Lack of knowledge Not the usual standard of care	Counterintuitive care	The provision of care which was contrary to normal practice and the evidence base about what works in preventing complications.
2	Large number of deaths Horrible Indiscriminate Burden Early death Anger Tears Not part of the team Away from normal support Not what I am used to Unexpected	Volume of death Tears and anger Support	Tough days	Days characterized by loss where several people die unexpectedly and where deaths go against the natural order in that they occur “before their time”
3	Giving up No regrets Family access Not the care I am proud of Sudden Unexpected Absence of family Personal Protective Equipment (PPE) as a barrier	Not proud Family access	Not the usual standard	The provision of care which conflicts with the practitioner's perception of what good care is. Or where care fails to meet the accepted professional standard.
4	Fear Vulnerabilities Feeling safe Concern about family Worried about PPE Availability of PPE Out-of-date PPE Frightened Anxious	Infection risk Fear and Anxiety Self and family	Personal and Family Safety	Concern about infection of self or family members because of exposure to a pathogen or because of inadequate or defective PPE
5	Tensions Tired Frustration Anger Struggling Scared Anxious Cover up Insomnia	Burnt out Struggling Pretending to be okay	Consequence of moral distress	The impact caring for Covid-19 patients during the pandemic had on individual practitioners and the wider team in terms of physical symptoms, social and psychological distress.

## Results

### Sample Characteristics

The sample was drawn from a single critical care department in the North East of England. The sample consisted of 10 registered nurses, all female with a mean age of 29.5 years (SD = 8.71), and two final-year female student nurses with a mean age of 21 years (SD = 0) completing their internship 21-week placement. The 10 registered nurses had worked in critical care for a mean of 6.25 years (SD = 8.01). In addition, the sample included two allied health professionals, one male and one female, with a mean age of 39 years (SD = 11.31) who had worked in critical care for a mean of 18 years (SD = 11.31). Finally, three consultant medical staff, two male and one female, with a mean age of 44.3 years (SD = 1.15) had worked in critical care for a mean of 10.5 years (SD = 0.70).

The interviews revealed detail of the source of moral distress from concerns about personal and family safety related to the infective nature of the virus, a lack of confidence about Personal Protective Equipment, as well as a high number of death and concerns about care delivery. The consequences for participants and their colleagues were also articulated. Table 2 details how the themes were defined and refined through the process of reflexive thematic analysis. The table provides details of initial coding and the development of initial themes through a process of identifying the five themes reported in this paper.

1. *Counterintuitive care*

One of the most significant sources of moral distress in the early stages of the pandemic was the presentation of the patients and the counterintuitive nature of the care guidelines which had been issued to clinicians.

*There was a lot of stupid guidance coming out about things like not humidifying circuits. We normally would do that but infection control had deemed that would aggravate droplet spread and that guidance was against everything we had seen and done over decades in intensive care. We followed the guidance and saw lots of complications we hadn’t seen for years. So we changed the care to what we know was best practice.*

Participant 11 - Medical

*Nowadays, when you deal with patients you are usually dealing with things you have seen many times before and you know what you are doing. I don’t think any of us really felt like we knew what we were doing to start with, you know it is quite unsettling. You know you have an expectation of how the course of the disease is going to go but this was so difficult and I think it is more upsetting actually when patients started to die. We didn’t really have an expectation of how and when that was likely to happen to start with, so I found it a bit tough. A lot of patients died after 2 weeks and which is quite unusual for critical care. Some of them died very suddenly. So that was a bit odd. But once you get a bit more confident with the natural history of the disease and how things play out it was easier to rationalise but overall the uncertainty was hard.*

Participant 8 - Medical

*Patients with Covid didn’t follow the traditional critical care trajectory. Normally patients don’t stay in critical care very long but the people with Covid were in 2-3 weeks, sometimes longer, before they started to die or improve.*

Participant 6 - Nursing

2. *Tough days*

Participants felt that the tough days when patients they thought we improving suddenly deteriorated and died or when there was a high number of deaths were particularly challenging for them emotionally and psychologically. The tough days were very tough for staff who had been redeployed to help as they felt they needed the support of familiar team members.

*I had come to help and didn’t feel part of the team and on the day when we had a large number of deaths before lunchtime. I remember that the NHS Trust management had decided that everyone needed more support because it was a horrible day, it was the worst day we had had in a long time. So the management came up and was on the unit but obviously not in the Covid part. They did a supported de-brief and there were tears because of the deaths, it was really emotional. I said to the staff I need to go and find ‘my team’ if that's okay. I need to go and find them.*

Participant 1 - Nursing

*I think the sheer number of deaths was hard. I had worked in surgery and people didn’t die that often and I did not have to do a lot of end-of-life care. We had daily deaths and it was quite tough. Also the number of younger people dying when it is normally people who are very frail with lots of comorbidities.*

Participant 2 – Allied Health

*I felt so angry for this poor family and how much they had suffered. There was just no rhyme or reason to it. It wasn’t as if people had made bad choices, that had impacted on them. It just happened and impacted me so significantly. I remember this poor lady was on her own and I just wanted to be with her, and I wish I could hold her hand. I promised her that I was with her husband and that I held his hand. He had no belongings because he had been transferred from a hospital 50 miles away. The only thing he had was a Help for Heroes band around his wrist. I remember thinking he must have been passionate to wear something like that. I said to his wife I have put that in a bag and will send it to you but don’t open it for 72 hours because of Covid. I said I will send it and she thanked me. But it was just the loneliness of all of the people.*

Participant 1 - Nursing

*We were dealing with the emotional burden of relatives who could not see their loved ones and that was challenging. We were having to be like a family because they could not come in and visit. They were using FaceTime and when people were dying we were holding their hands and offering comfort… It was difficult trying to stay strong for them but I sometimes had to look away because I had tears streaming down my face when people finally died.*

Participant 16 - Nursing

3. *Not the usual standard*

Participants expressed concerns that they were delivering care that they regarded as sub-standard when compared to the care delivered outside the pandemic.

*I remember caring for this man who couldn’t tolerate the mask anymore and was dying. I was holding back tears and thinking to be professional.. I called his wife and she said you’ve got it wrong, he wouldn’t give up. He wouldn’t give up, not my .. and she said his name. She said I am on my way. When she arrived I quickly started putting PPE on her. I said I don’t want her to have any regrets and he hasn’t got long left. She came in and held his hand and he took two more breaths and then passed away and she just broke down. I remember thinking, this poor man and his wife, that is not how death should be and it's not the death I pride myself on. Everything was so sudden and unexpected that you had little control over it.*

Participant 1 - Nursing

4. *Personal and Family Safety*

Participants described how they were concerned about the availability and quality of Personal Protective Equipment as well as concerns for the safety of family members during the early part of the pandemic.

*I probably felt quite safe in that I am still relatively young and healthy… I guess I worried more about family members. Later in the pandemic, I started to worry when younger people who were relatively healthy before they caught the virus started to be admitted.*

Participant 3 - Nursing

*The strategies which were designed to preserve PPE weren’t necessarily good for staff because they had to delay breaks and couldn’t get downtime when it was needed. When you’re trying to deal with Covid and you’ve got additional stressors like a shortage of gowns and masks which are out of date it isn’t good.*

Participant 17 - Nursing

*At first I was very frightened and while you’ve got all the PPE that's available you feel concerned about going home and you know taking things back to your family.*

Participant 12 – Allied Health

5. *Consequences of moral distress*

Participants described how they struggled and many of them soldiered on “keeping up appearances.” Several described the psychological impact of working in critical care during the pandemic as well as the physical manifestations of fatigue.

*You can see the tensions rising in the department sometimes because people are fatigued and tired and they get frustrated.*

Participant 4 - Nursing

*I think people did their best and a lot of people struggled. Emotionally you are trying to manage a situation where emotions are running high, people are scared themselves and scared of taking the infection home.*

Participant 5 – Nursing

*When you’re a senior member of the team you’ve got to sort of keep up appearances. Even when you’re not really all right. If you start to crumble then that's not good for the rest of the team.*

Participant 5 - Nursing

*I started not sleeping. When the next wave I ended up not able to sleep in my bedroom. Every time I went to bed – which should be where I perhaps feel safest I couldn’t sleep. I felt like I was drowning and that duvet was suffocating me. So I ended up sleeping on the sofa because I couldn’t go into the bedroom.*

Participant 7 – Nursing

## Discussion

While much is known about the sources of moral distress in critical care settings outside of a pandemic this study provides in-depth insight into the nuanced differences between the normal stressors and those which occurred during COVID-19. The suggestion that counter-intuitive care and the fact that COVID-19 patients did not follow a typical critical care trajectory were interesting. Counter-intuitive care appeared to be the result of national guidance on managing patients. This seemed to be the opposite of normal care such as the humidification of ventilation circuits. Staff quickly reversed the approach as they started to see complications arising from the national guidance. In many ways, the moral distress associated with counter-intuitive care was similar to the distress which arises from delivering care which the practitioner does not believe in the best interests of the patient. Such care delivery can give rise to moral distress when the practitioner believes the care is futile (Mobley et al., 2007). Futile care is often the result of conflict between the clinicians and the patient's family members and this in turn can create moral distress (Hiler et al., 2018). There are of course subtle differences as the counter-intuitive care involved the practitioner delivering care they felt was potentially harmful rather than simply not in the patient's best interests. Another significant issue is that futile care and family conflict is relatively rare whereas counterintuitive care, at least in the early stages of the pandemic was widespread because of the guidance about non-humidified ventilation circuits. While research has been conducted to explore moral distress arising from patient safety concerns this has to date been specifically related to dysfunctional teams or safe staffing levels (Berhie et al. 2020; Papathanassoglou et al., 2012).

While the studies undertaken by Hines et al. (2021) and Romero-Garcia et al. (2022) acknowledge both resource issues and patients and their families as sources of moral distress neither study identified the natural history of Covid-19 as a source of moral distress during the pandemic. Uncertainty in terms of disease progression appeared to be a new source of moral distress. This was often associated not only with concerns about whether the treatment and management were appropriate but also with late death after a prolonged period of care. Several participants remarked on rapid deterioration when the usual trajectory was patients either not responding to treatment and then dying quickly or people getting better and being transferred to other wards. Research on uncertainty and moral distress has tended to focus on end-of-life care, assisted suicide, and following advance directives (Dorman et al., 2020; Rawas, 2019; Ribeiro et al., 2020). More widely the notion of uncertainty in clinical decision-making and treatment in critical care has been explored in more detail with a focus on deciding preferred care outcomes and exploring trials of treatment (Beil et al., 2022; Higginson, et al. 2016).

Critical care nurses and staff are no strangers to dying patients (Boissier et al., 2020) and some of the participants described the type of normal death they would be proud of where family members are supported through the process. During the pandemic, it was not possible to deliver this care and staff faced the extra burden of being family members holding a patient's hand and staying with them. The volume of deaths was different as well, with participants describing tough days with a large number of deaths or people dying just when they thought they had started to improve. Providing end-of-life care is a known source of moral distress in critical care often because of conflicts between the wishes of the patient's family and the perceived best interests of the patient (St Ledger et al., 2013). Romero-Garcia et al. (2022) identified that the absence of family members was the single biggest source of moral distress in their survey of Spanish intensive care staff. Romero-Garcia et al. (2022) describe how staff experienced problems alleviating their own emotional suffering and distress when watching patients die alone. The participants in this study described in detail the lengths they went to in order to support families using video conferencing facilities or supporting people to be at the patient's bedside in the last few minutes of their lives. Not being able to provide the usual standard of care was also a source of moral distress this was usually associated with what participants felt was a good standard of end-of-life care for the patient and their families.

Another new source of moral distress highlighted in this study related to concerns about family and personal safety. Concerns about moral distress and a lack of personal protective equipment during the pandemic have been widely reported (Altman, 2020; Guttormson et al., 2022; Lake et al., 2021). According to Lake et al. (2021), the lack of personal protective equipment and concerns about infection caused occasional moderate moral distress. Such concerns were more widespread during the early phases of the pandemic because of a global shortage of personal protective equipment and while the transmissibility of the infection was unclear (Hoernke et al. 2021). Such concerns relate to personal safety and concerns about becoming infected with the virus. There is a dearth of research which highlights concerns about the transmission of infection to the nurse or practitioner's own family members. Participants highlighted concerns about older family members or those with long-term conditions. While this source of moral distress is related to infections it is important that globally there are more than 100,000 infection outbreaks with many requiring hospitalization (WHO, 2019).

The participant's descriptions of the consequences of moral distress illustrate the huge toll this has taken on people. Many reported feeling burnt out and many experienced low mood, depression, and flash-back episodes suggesting significant moral injury and other psychiatric problems. At the same time, senior members of the team described having to “keep up appearances” to provide reassurance to the wider team. Johnson-Makiya (2016) explored the relationship between moral distress, leadership integrity, and turnover intent. The researcher suggests that leadership integrity is the observation of behaviors which match someone expressed values and beliefs. Leadership integrity acts as a buffer for moral distress providing staff with reassurance that colleagues in leadership positions are watching out for them during times of stress and difficulty (Johnson-Makiya, 2016).

## Strengths and Limitations

This is an in-depth study exploring sources of moral distress and while there are some similarities with previously published work on moral distress in critical care there are a number of differences associated with high patient volumes with a life-threatening infection. The research explores moral distress from the perspective of multiple practitioners spanning nursing, medicine, and allied health professions. The research is limited as it was carried out in only one critical care department between the first and second (UK) waves of the pandemic. The study suggests that the frequency of exposure to moral distress, for example, high number of deaths on a single day plays a part but the study does not measure the frequency of exposure over a specific time period. In addition, the retrospective nature of naturalistic inquiry which involves participants constructing meaning may have altered perceptions over time as they may have rationalized and thought through the issues they experienced. While the results of this qualitative research are not generalizable to the wider population they may be transferable to practitioners in a similar context (Ritchie & Lewis, 2003).

## Implications for Practice

The findings suggest that during outbreaks of infection, especially with new and emerging infections, staff can experience considerable anxiety about their own and their family's safety. This can be further compounded by uncertainty about patient management and treatment approaches. From the outset systems of support such as de-brief and group support should be implemented from the outset of any infection outbreak. Such approaches go some way to providing staff with initial support and an opportunity to explore morally distressing situations.

The research also highlights the value of leadership not only in determining the approach to patient care but also in supporting colleagues through their words and behaviors. It is important to remember that leaders need support and they should have opportunities to engage in de-brief where they can share anxieties and concerns outside of the team they lead.

Finally, where possible it is important to counterbalance infection prevention and control with appropriate family access (wearing personal protective equipment) and with care delivery. This would enable family members to be present in people's final hours, enable appropriate support to be offered and lessen the moral distress associated with perceived less-than-optimal care.

## Conclusion

This study explored the potential sources of moral distress during the pandemic and the meaning that practitioners attached to their experiences. There were some similarities with the sources of moral distress in critical care which occur outside of a pandemic. However, the frequency and intensity of the experiences are likely to be different during a pandemic, with staff describing high volumes of deaths without family members present. In addition, new sources of moral distress related to uncertainty, counter-intuitive care and concerns about the personal and family risk of infection were identified. Given the volume of distressing incidents earlier use of group support, de-brief and specific support for leaders who may feel unable to discuss issues with the teams they are leading would have been beneficial.
